# Lessons from Africa: developing a global human rights framework for tuberculosis control and prevention

**DOI:** 10.1186/s12914-014-0034-7

**Published:** 2014-12-03

**Authors:** Tracy Slagle, Mehdi Ben Youssef, Golda Calonge, Yanis Ben Amor

**Affiliations:** The Earth Institute, Columbia University, New York, NY USA; The School of Social Work, Columbia University, New York, NY USA

**Keywords:** Tuberculosis, Drug-resistant tuberculosis, Framework convention, Human rights

## Abstract

**Background:**

Tuberculosis is a highly contagious disease, and there has been a rise in recent years of drug-resistant cases no longer responding to standard treatment.

In order to address this threat and contain possible transmission of drug-resistant cases, some countries have taken strong action, including the compulsory detention of non-adherent drug-resistant patients. These measures have been strongly criticized by human rights advocates, and they raise the question of how to legally protect both citizens and the community.

**Discussion:**

Following discussions with National Tuberculosis Programs in Africa (the continent with the highest incidence rates of tuberculosis worldwide), we show that of all the countries surveyed, all but one (Swaziland) had either no specific policy addressing tuberculosis, or only general policies regarding public health applicable to tuberculosis. Six countries also reported having policies that address non-adherence to treatment with containment (isolation in health facilities or incarceration), but laws are not adequately enforced. If the international community wants to effectively respond to the threat of tuberculosis transmission, there is a need to go beyond national tuberculosis policies and to implement an international framework for tuberculosis control, inspired by the Framework Convention on Tobacco Control, a key model for future public health treaties that address global burdens of disease. The framework, for which we clarify the conditions and procedures in this piece, would define the rights and responsibilities of the different stakeholders involved: patients, doctors, pharmaceutical firms and public authorities. To facilitate the governance of the national obligations under the Convention, a coordinating body should be set up, under the leadership of the World Health Organization and the Stop TB Partnership.

**Summary:**

Successfully implementing policies for tuberculosis that simultaneously address patients’ rights and communities’ wellbeing will have positive implications for those affected by the disease and serve as a basis for other global health conventions to truly ensure the global right to health.

## Background

### Overview of tuberculosis

Tuberculosis (TB) kills more than 3,500 people each day worldwide, leading to approximately 1.3 million deaths yearly [1]. One third of the world’s population is infected, and 8.6 million new cases of active TB are estimated to occur around the world each year. TB is highly contagious: each person with active TB, if left untreated, will infect an average of ten to fifteen people annually [2].

Most cases of TB are drug-susceptible, therefore curable. However, there has been a rise in recent years of drug-resistant cases that no longer respond to standard TB antibiotics. Multidrug-resistant TB (MDR-TB), estimated at 450,000 cases a year worldwide [3], or the virtually incurable extensively drug-resistant TB (XDR-TB), have been detected in every part of the world [4]. In an effort to respond to this threat and contain possible transmission of drug-resistant TB within communities, some countries such as Kenya and South Africa have taken strong actions, including the compulsory detention of non-adherent MDR-TB and XDR-TB patients [5]. In Kenya, two non-adherent TB patients were deemed a public health threat to their communities and incarcerated for several months in 2010 [6]. In 2012 in the Unites States, a California man infected with tuberculosis was charged and jailed for not taking his TB medication [7]. Such measures have been strongly criticized by human rights advocates and international organizations that denounce a violation of human rights for patients who are punished for the inadequacy of the health systems [8].

### The World Health Organization and International Public Health Frameworks

International agencies including the World Health Organization (WHO) and the Center for Disease Control and Prevention (CDC) have issued policy recommendations on how to better balance global public health and individual rights in managing TB [9-11]. The WHO specifically has the ability to disseminate regulations and agreements on international health^a^, including standards related to “quarantine requirements…to prevent the international spread of disease^b^,” and the ability to oversee any national health legislation promulgated in each member state’s jurisdiction^c^. The WHO is also in a unique position as an international organization: per its Constitution, it can draft conventions that are binding on all signatory member states^d^.

However, the WHO has only asserted this authority once, in the Framework Convention on Tobacco Control (FCTC) of 2003 [12,13]. While international guidelines on TB prevention, care and control exist [10], there are no legally binding standards to ensure that states are actually implementing the required guidelines. This raises the question of how to create enforceable guidelines and policies that will protect citizens and the international community. Can a TB control framework be implemented by different countries under the auspices of the WHO and the Stop TB partnership? Is there even a need for an international legal framework specific to TB? How would this proposed framework balance rights of the individual and the rights of the collective, or more broadly, take into account public health concerns?

### The right to health

Since the 20th century, human rights have increasingly become a central preoccupation of the international community [14]. Several instruments were adopted to codify and protect human rights, including the Universal Declaration of Human Rights (UDHR) of 1948 [15], and the International Covenant on Economic, Social and Cultural Rights (ICESCR) of 1966, which recognizes in its 12th Article “the right of everyone to the enjoyment of the highest attainable standard of physical and mental health” [16]. Currently, every state in the world has signed on to a treaty or covenant that asserts the right to health [17]. Both the ICESCR and the International Covenant on Civil and Political Rights (ICCPR) [18] of 1966 further enumerate and define the rights within the UDHR, and place enforceable obligations upon national governments to *respect, protect* and *fulfill* each human right for the citizens of their country. Similarly, the UN Committee on Economic, Social and Cultural Rights published “*General Comment No. 14”* in 2000, which defined what the right to health implies, and outlined obligations for each state: a state is required to *respect* the right to health by abstaining from actions that limit or make unequal access to “preventive, curative and palliative health services [19]”. A state also will *protect* the right to health by “ensuring equal access to health care and health care-related services” [19]. Finally, a state is required to *fulfill* the right to health by establishing “provision of health care… and to adopt measures against environmental and occupational health hazards and against any other threat as demonstrated by epidemiological data [19]”.

Beyond the ICESCR, ICCPR and *General Comment 14*, there have been additional attempts at reaffirming and defining not only the right to health but also corresponding national and international obligations, in international human rights treaties such as the Convention on the Rights of the Child [20], regional charters such as the African Charter on Human and People’s Rights [21], and in national constitutions such as that in South Africa [22]. The Siracusa Principles, adopted in 1984 [23], have established conditions and criteria to be addressed when restricting human rights in order to avoid abuses, such as the necessity to take decisions in accordance with national laws and ensure those decisions are neither disproportionate nor arbitrary [24]. Currently there is also a movement supported by a broad base of key players from the fields of human rights and public health, with public supporters such as UN Secretary General Ban ki-Moon and UNAIDS Executive Director Michel Sidibé, to create a framework convention on global health, outlining in legally binding terms the obligations of states and related parties with regard to the right to health and all it entails [25].

However, these existing international covenants have not yet specifically laid out a framework for infectious diseases that have truly global repercussions, such as tuberculosis. While the Siracusa Principles for instance offer acceptable guidelines, they also have limitations. As a result, they have not been effectively implemented in any country that originally signed on to their adoption. This is due mainly to three reasons. Firstly, the Siracusa Principles were produced by the Economic and Social Council of the United Nations (ECOSOC), which make them mere recommendations. Indeed, the type of ‘soft law’ contained in the Siracusa Principles has no legal value and is not binding. Secondly, the Siracusa Declaration is not specific to global health but more specifically targeted to the derogation of civil and political rights, and therefore lacks precision in this regard. Finally, the Principles refer to the necessity to comply with national laws, which, specifically for TB do not yet exist, as explained in more detail below. Therefore it appears clearly that there is a need to go beyond the Siracusa Principles.

In addition, while we are in full support of a framework convention for global health, we are hesitant in expecting such a broad framework to solve the problems that a trans-boundary disease such as TB poses to global health. In fact, the inclusion of how articles and guidelines pertaining to specific diseases should be included, if at all, is an outstanding question posed by the Joint Action Learning Initiative on National and Global Responsibilities for Health, a key thought supporter for such a framework convention [26]. We are therefore advocating in our opinion paper that while a framework convention for global health is necessary, that either explicit guidelines for state actors and private sector companies specific to TB must be included, or either a simultaneous process to negotiate a framework convention on TB must be done, to serve as a model for future framework conventions on specific diseases, such as malaria, HIV/AIDs, non-communicable diseases, and others.

Currently, we find ourselves in a strategic time, in that the momentum from the Millennium Development Goals that brought renewed worldwide attention to health issues such as TB is now transitioning to a new post-2015 era where international goals and targets for public health have yet to be defined. The global health and human rights communities must seize the opportunity to introduce on the international stage a framework convention that either explicitly includes TB, or one specific to only TB, in order to capitalize on this momentum.

## Discussion

### Tuberculosis legislation and the right to health in Africa

We focus the discussion in this section on Africa because the continent suffers from the highest incidence rates of TB worldwide, and most TB high burden countries are located on that continent. In the past few years, there has been major progress towards global targets for reduction in the burden of the disease. The 2015 MDG target of halting and reversing TB incidence has been achieved, with TB incidence falling globally for several years in all 6 WHO regions, including Africa. However, the African region, home to 11% of the world’s population, still has approximately one quarter of the world’s cases (255 incident cases per 100,000 on average, more than double the global average of 122) and the highest rates of cases and mortality relative to population. While prevalence and mortality rates are declining in all 6 WHO regions, the African region is not currently on track to meet the 2015 MDG target for prevalence or mortality^e^. The proportion of TB cases co-infected with HIV is also highest in the African region. Overall, 37% of TB cases were estimated to be co-infected with HIV in this region, which accounts for 75% of TB cases among people living with HIV worldwide.

African healthcare systems are particularly constrained in their ability to respond to the threat posed by tuberculosis due to limitations such as lacking adequate facilities, trained personnel, reliable drug supplies, laboratory capacity and most importantly, access to steady funding sources. Although the Global Fund to Fight AIDS, Tuberculosis and Malaria (GFATM) and the President’s Emergency Plan for AIDS Relief (PEPFAR) have donated large sums to help address Africa’s health problems, most of the funding has been earmarked for HIV, with a lesser financial focus on TB. In 2013, analyses were conducted in the context of estimates of funding needs and funding gaps for a full response to the TB epidemic. The total funding required in all low- and middle-income countries was estimated to be US$ 8 billion in 2015, compared to US$ 6 billion in 2012. The Global Fund accounted for 64% of all donor funding reported by countries during the decade 2002–2011. Despite growth in funding from domestic and international donor sources, National TB Programs (NTPs) were not able to mobilize all the funding that they estimated to be needed for TB prevention, diagnosis and treatment. Funding gaps persisted and increased from US$ 257 million in 2002 to US$ 563 million in 2011 to US$ 1 billion in 2013, with the African region accounting for 48% of this funding gap.

In order to adequately address the threat of TB, the WHO has taken key steps, particularly in recent years, by promoting various interventions, including the use of a rapid, molecular-based technology for diagnosis of TB [27], increased investments in laboratory services, focus on preventive therapy for HIV-infected patients and surveillance of drug resistance. In line with the elaboration of a post-2015 development framework, the WHO also began in 2012 the process of developing a post-2015 global TB strategy. The draft strategy includes the goal of ending the global TB epidemic by 2035, with corresponding global targets for major reductions in TB cases and deaths by 2035. Achieving the proposed targets is based on three strategic pillars: integrated, patient-centered TB care and prevention; supportive systems; and intensified research and innovation.

**Figure 1:**
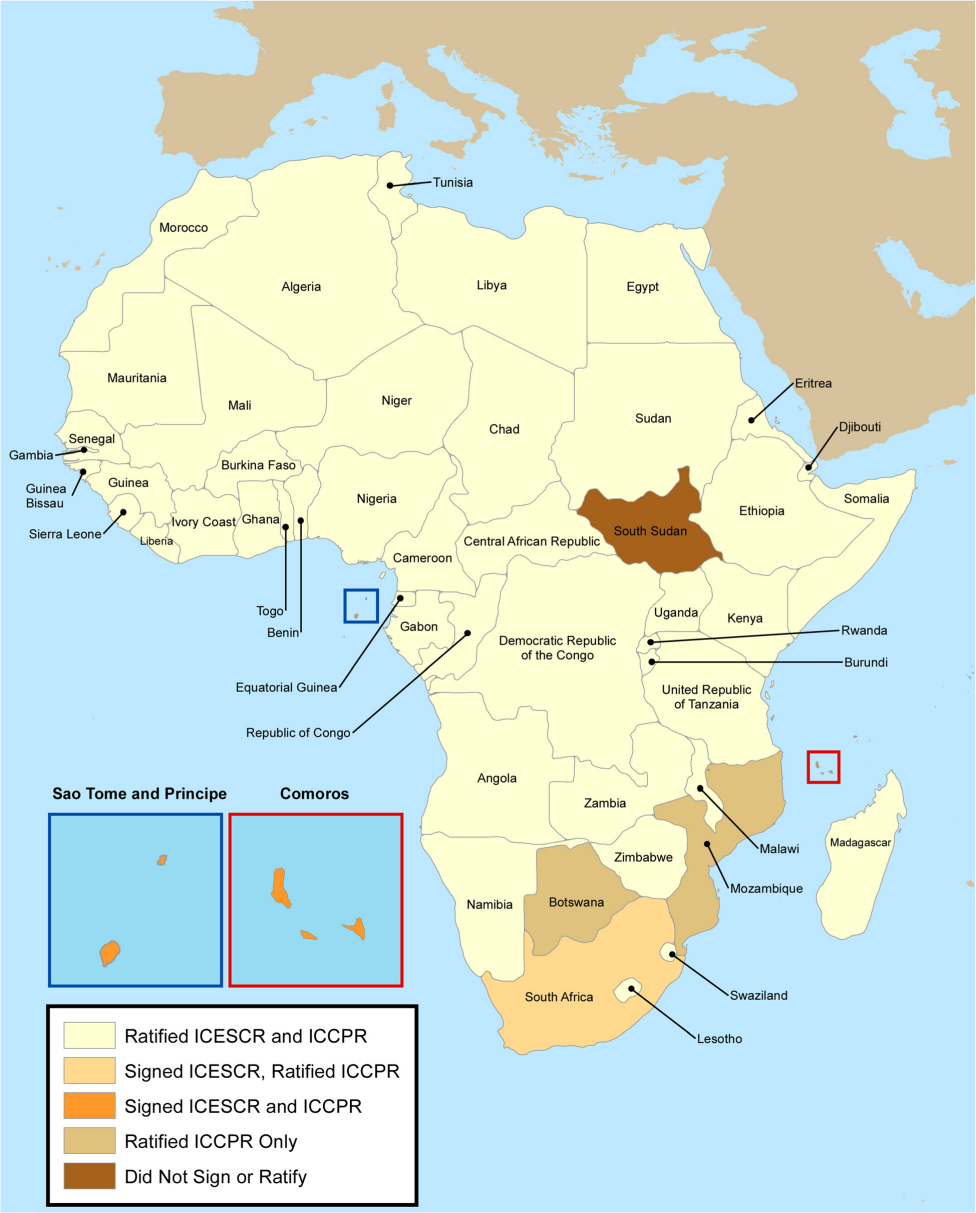
**African states that signed or ratified the International Covenant on Economic, Social and Cultural Rights (ICESCR) and the International Covenant on Civil and Political Rights (ICCPR).**

The WHO first declared TB a “Global Emergency” in 1993 and since then, significant efforts have been undertaken to control the disease. This strong focus on TB control and prevention has led to substantial gains worldwide, including in Africa, demonstrating that with the adequate investment of resources and political will, the disease can be curbed. However, despite the major gains highlighted earlier, the curable disease still claims close to 1.3 million deaths every year. What is needed moving forward is a systematic and rigorous effort that will simultaneously solidify the gains in TB control since the early 1990s while also expanding to other diseases such as HIV or malaria by strengthening health systems generally. The experience in Africa demonstrates why an international TB control framework is necessary and timely in the post-2015 context. To put the situation in the context of human rights, the vast majority of African countries have signed on to both ICESCR and ICCPR covenants (Figure 1). Fifty-one African countries have agreed to the terms of the ICESCR by signing the covenant, while 48 states out of 55^f^ have ratified it. All of the states colored yellow in Figure 1 have therefore declared to the global community that they will *respect*, *protect*, and *fulfill* the right to health for their citizens.

Given the importance of the right to health and its corresponding obligations across the African continent, in these same states where incidence of a trans-boundary disease such as TB is highest, special attention must be made to controlling this disease in order to ensure the right to health for all.

**Figure 2:**
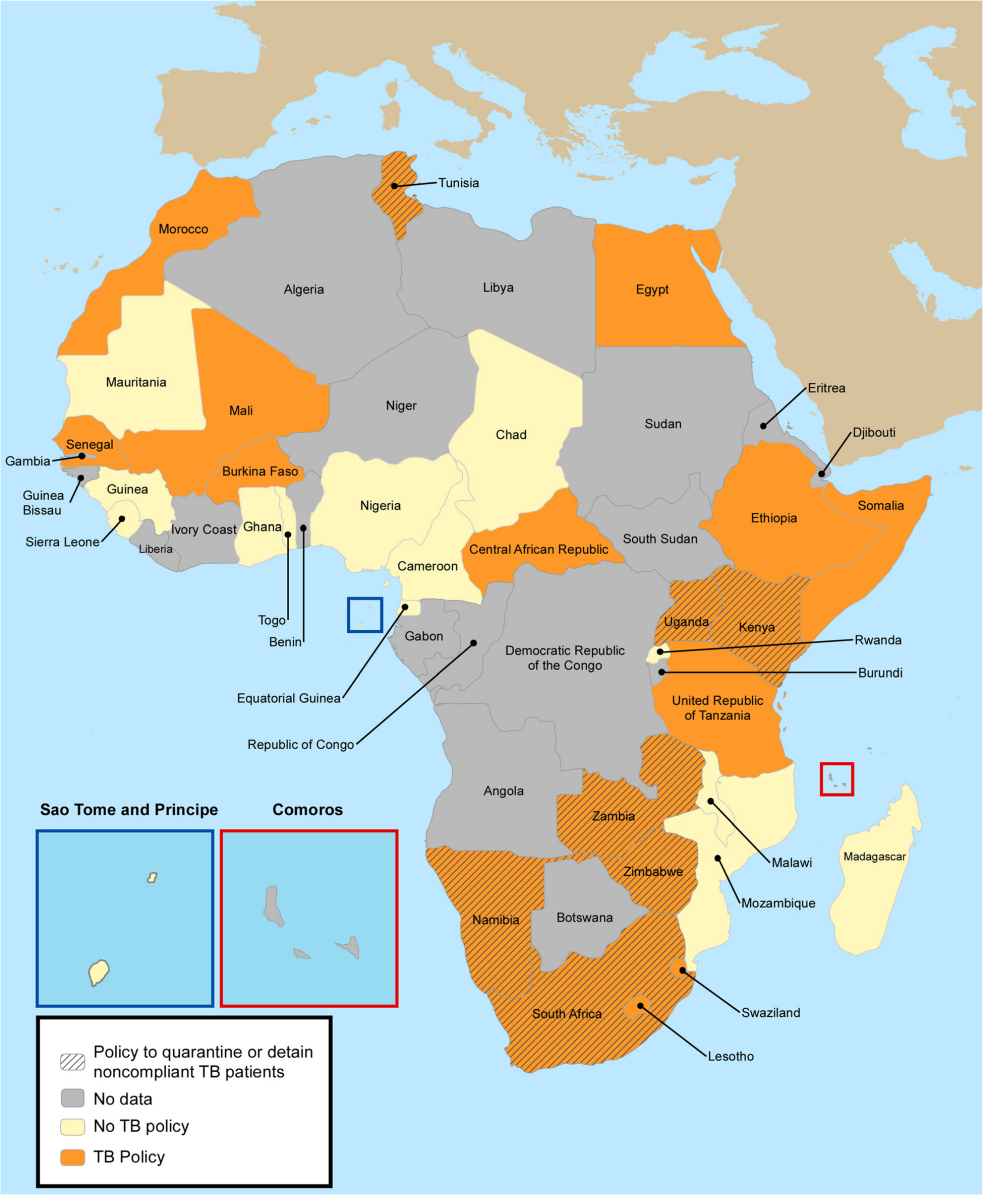
**Tuberculosis legislation in African states.** Specific tuberculosis-related legislation was mapped in 34 states in Africa that responded to our written survey (out of 54). Based on responses from representatives from National Tuberculosis Programs, states were classified as either having no tuberculosis policy (yellow), or general policies regarding public health and/or communicable diseases that could be applied to tuberculosis (orange). The only country with a specific policy on tuberculosis (Swaziland) was included in the latter category (orange). Finally, countries that adopted policies to quarantine or detain noncompliant tuberculosis patients appear in dashed.

In research for this paper, we examined the commitment of African states to implement specific health policies for TB by conducting a brief survey of heads of National TB Programs (NTPs) in Africa. Written surveys were sent via email to 54 NTPs and follow up phone calls were organized when needed. Some officials declined to answer or were unavailable during our investigation. We gathered information for 34 countries. Figure 2 illustrates whether African countries have explicit policies addressing TB. Of the NTPs interviewed, all but one (Swaziland) indicated that their country had either no policy addressing TB, or only general policies regarding public health and/or communicable diseases that are not specific to TB, but included TB policies. Six heads of NTPs (Kenya, Namibia, Uganda, Tunisia, Zambia and Zimbabwe) reported having policies that address non-adherence to DOTS (Directly Observed Therapy – Short course is the WHO-approved treatment strategy for TB [28]) with containment (isolation^g^ in health facilities or incarceration^h^). Most respondents agreed that containment laws were not effectively enforced.

The absence of clear policy surrounding TB patients’ containment illustrates the challenges governments face with regard to isolating the illness while still protecting individual freedoms. Although non-adherence may represent a personal choice for individuals and not simply the failures of the health system, the contagious nature of TB makes one individual’s non-adherence potentially life-threatening for communities. Therefore, it is imperative that countries organize or strengthen their enforcement of TB policies in order to protect their populations.

In our survey, only Swaziland’s NTP reported that its government’s legal framework included national policies specific to tuberculosis^i^. Interestingly, this specific legislation does not include a section on containment for non-adherence. However, if the international community wants to effectively respond to the global threat of tuberculosis in a coordinated manner and adequately help countries, there is a need to go beyond the basic international human rights covenants and limited attempts at national TB policies, that have so far failed to reach their goals, and to implement an international TB control framework that would assist countries in defining appropriate policies and ensure that patients’ right to health is respected. The basis for designing and implementing such a TB control framework exists given the commitment of these African states to protecting the human rights of their citizens, including specifically to *respect*, *protect*, and *fulfill* the right to health. In addition to governments, civil society organizations have also made a substantial positive impact throughout the regions of Africa [29], not only by raising awareness and improving uptake of services, but also importantly by holding governments to account. Thus now is the time for action as we are entering the post 2015-era, particularly given the increasing burden of drug resistant TB, as well as the lack of national TB policies that protect the human rights of citizens, while also controlling the spread of TB and MDR-TB.

### Public health and international human rights: the case of the FCTC

As noted above, the idea of an international framework for global health has been proposed [25,30] in an effort to reduce disparities and provide better coordination among national and international stakeholders. Inspired by the FCTC, this would certainly be notable progress as the first treaty to provide guidelines for international regulation of global health, and should start by addressing the critical and trans-boundary disease burden of TB. We believe the first such legal framework should be focused on tuberculosis because it is a respiratory infection that is highly contagious, and transmission occurs while patients are passively interacting with their surroundings simply by breathing, rendering prevention difficult [2,31].

There are two potential ways to realize this: the first is by ensuring that the proposed framework convention on global health includes disease specific articles and rules on actions that states, the private sector and other relevant parties (as defined below) must take. This would appear to be an attractive option, as TB is but one area where an international human rights framework could prove beneficial in motivating states actions. There is also an advantage in strengthening health systems as a whole. However, we do not propose that TB be given cursory acknowledgement in a larger framework convention in a way that does not include specific responsibilities and guidelines such as those suggested in the following section. Therefore, in our second and preferred option, we would propose that, using the model of the FCTC, and building upon the international conversation for a framework convention on global health, that a framework convention specific to TB be negotiated. This could be achieved either simultaneously as a framework convention on global health, or separately by starting with a framework on TB where other diseases specific targets could be built in subsequently, either way issued by the WHO as a legally binding convention. This latter option would prevent overburdening signatory countries with a series of reforms for various diseases that, while complementary, could possibly lead to a financial strain on existing health systems. However, even in the context of a framework convention specific to TB, scarce resources would not just be committed to a single disease, possibly at the detriment of other health problems. We believe that the implementation of the FC would not only focus on strengthening access to TB services, but also lead to health systems strengthening as a whole. For instance, better laboratory infrastructure for TB diagnosis will also benefit access to adequate microscopy-based diagnosis to tackle diseases such as malaria or shistosomiasis. Improved patient management, particularly around adherence and management of lost-to follow up will also benefit other patients with chronic diseases, infectious or not, such as HIV or diabetes.

As noted above, the WHO can issue binding treaties, and the FCTC was a significant first example of this important authority. The FCTC requires that states implement legislation that protects their citizens from deleterious health effects such as secondhand smoke, and design national programs that target a reduction in nicotine addiction. The FCTC thus has implications for member states, as well as for private sector companies that sell tobacco internationally. Though the FCTC is not a human rights treaty, it includes allusions to human rights. In keeping with human rights norms, it also places obligations on states with regard to tobacco control that are inherently a component of a state’s responsibility to *respect*, *protect*, and *fulfill* the right to health. As of September 19, 2013, 177 parties were bound by the FCTC, representing 88% of the global population, including 47 states in Africa [32].

The FCTC model is therefore key for future public health treaties that address global burdens of disease. The FCTC should serve as an aspirational model. However, we do not recommend transposing the FCTC in its entirety to develop the new framework since there would be inherent differences between the two, mainly that the FCTC addresses a risk factor while our proposed framework focuses on a disease. Consequently, the measures related to a disease such as TB must be more specific than currently enforced. We believe such a framework convention as the FCTC can be used as a model of international cooperation with corresponding obligations on member states and the private sector to protect the right to health for individuals in a binding legal framework.

While it is necessary to note that defining, drafting and negotiating such a convention can be a long and arduous process, the reason we look to a framework convention for TB rather than existing guidelines issued by the WHO such as the *Guidance on Ethics of Tuberculosis Prevention, Care and Control* [10], is that guidelines are just that – suggestions on how a state or private sector company or other related party should behave, with little enforcement power, and often not backed by financial or other resources as incentives to motivate institutions to support their implementation at the national level. As the WHO notes in the above referenced *Guidance,* there are responsibilities on state actors to prevent and manage TB, which are generally accepted and well known, but, as they state, “TB has not yet been eradicated mostly because these responsibilities have been neglected” [10].

Finally, such a framework ‘convention with definitive international standards for TB prevention, care and management, grounded in human rights, would benefit all state parties involved. Controlling TB is not just in the interest of countries where the disproportionate burden of disease lies. With an airborne disease such as TB, and the ease of traveling from one country to another with any manner of illnesses (including, as we’ve seen this year, with Ebola), controlling TB is in every country’s interests. And those countries with the highest burdens of disease would benefit from increased attention and support to this disease, including financial resources that are desperately needed (with more information on this below) [33]. Thus despite the resources and time that negotiating such a framework convention will require, establishing a set of enforceable and justiciable international responsibilities for states that would ensure a human rights-based approach to TB prevention, care and management, would be well worth the resources expended.

### An international legal framework for TB

The legal framework we are proposing would define the rights and responsibilities of the different stakeholders involved in TB treatment and control. Its objective is to protect communities and the human rights of TB patients [34]. Civil societies and social movements will be particularly critical in turning these rights commitments into a reality on the ground.

#### The patients

First and foremost, it is important to *protect* the right of the patients, in ensuring that they have access to equitable and consistent treatment. It is essential to provide them clear and complete information regarding the state of their disease and its possible evolution both in terms of health and legal implications. This could be achieved through counseling from healthcare providers, or distribution of informative brochures at the time of diagnosis. While these are already recommended in the national guidelines, they are not always enforced, particularly in rural settings in low-resource countries. Many patients, including in developed countries, have suffered from the lack of precision about their rights because of the absence or disparity of regulations [35]. A clearly defined legal framework would guarantee the fundamental right of all TB patients to information related to their condition and would allow them to comprehend their need for treatment.

The first step in successful treatment outcome is accurate diagnosis, and based on the most recent WHO report, close to a third of the expected cases of TB worldwide go undiagnosed [1]. These undetected TB patients unknowingly contribute to the spread of the airborne disease, and pose a threat to their communities. While the most commonly used diagnostic tool (sputum smear microscopy) is imperfect and can only detect on average half of the cases, new molecular-based tools for TB diagnosis are available, and are part of the WHO strategy for increased case detection [27]. Unfortunately, despite a scale up in recent years, these tools are not currently available everywhere and most health centers, particularly in Africa, still rely on microscopy. The framework should include a strategy for prevention and control benefiting the community that would involve sensitization (during market days, in schools, during community meetings) on the most common signs of tuberculosis, and promotion of early diagnosis, which should be paired with an improvement of diagnostic structures at health facilities. The community could directly be involved through the rollout of Community Health Worker (CHW) programs for door-to-door detection of TB suspects, and subsequent referral to health centers for diagnosis. Civil society organizations should particularly be involved and empowered to reinforce positive messages that TB is curable, and that treatment is free. Civil society organizations should also be mobilized to form treatment support groups within the community to assist TB patients throughout their six-month treatment. Furthermore, donors and governments acknowledge that civil society benefits populations by providing a measure of accountability. Institutional goals, such as transparency, are often cited as important reasons for having monitoring bodies that advance citizens’ interests [29].

Based on WHO guidelines, TB patients who do not take their medication for eight consecutive weeks or more are classified as “defaulters” or “lost to follow up”. In order to prevent this, a set of rules to ensure adherence and access must be undertaken. This can be achieved through community-based DOTS, or the use of new mobile-health technologies [36-38]. Given the enormous consequences of failed TB treatment, not providing quality and accessible treatment to a patient can result in a violation of their human rights. Additionally, it cannot be assumed that any failure to take the medication is solely a fault of the patient. Dr. Paul Farmer has completed ethnographic studies of TB DOTS adherence in countries such as Haiti, Peru and the United States, and has found that numerous times a patient deemed “lost to follow up” is a negative consequence of the public health system, rather than any malicious conduct and neglect on the part of the patient [39]. With that in mind, any international TB control framework has to take into account the issues patients face around access to treatment, and promote equitable access to effective curative treatments. This would effectively ensure the *fulfill* requirement of the framework.

If all efforts to follow up with patients have not been successful, including patient tracing through CHWs and attempts at counseling sessions, public authorities should be able to select home confinement^j^ with mandatory DOTS supervised by CHWs. If the patient persists in not respecting treatment requirements, public health authorities should be able to increase the stringency of their actions towards containment if they consider it to be a necessity to protect the community until the patient’s completion of treatment. Containment therefore serves the double purpose of ensuring completion of treatment while also preventing transmission in the community. A recent survey carried out by the Ethics Advisory Group from members of the International Union Against TB and Lung Disease showed that 83% of respondents supported involuntary incarceration as their preferred mechanism of containment [40]. However, we believe containment should be organized in public health facilities rather than prisons, as was unfortunately done in the case in Kenya in 2010. Not only would incarceration violate patients’ rights, they may also unnecessarily place at risk the cellmates of TB patients since infection control cannot be ensured in the prison environment [41]. In the home or public health facility, it is easier to protect against infection of others, and patients are more efficiently managed by healthcare professionals, rather than prison guards. However, we need to stress the need to provide health facilities that are appropriate for the treatment and care of patients who have an airborne disease such as tuberculosis (such as adequate ventilation). Examples of nosocomial^k^ transmission of TB and MDR-TB to healthcare staff abound [42-44]. At any time, on the basis of the patient’s response to treatment and their willingness to be adherent, these constraining solutions should be adjusted and the patient returned to a regular clinic-based DOTS.

It is essential with these measures to *respect* the right to health and guarantee a right to appeal for the patient [45]. Though it may be a challenge for local and national governments alike to provide adequate legal representation for their citizens regardless of socio-economic status, it is still an imperative piece of any TB control framework. To address this and ensure that containments are equitable and only required in rare circumstances where other methods have failed, additional mechanisms need to be built into the framework. The framework should empower NTPs to first monitor any requests for containment, and to ensure that each patient is adequately and fairly treated. The NTP must monitor the authorities and patients while under containment, and provide yearly updates to a coordinating body of representatives from among the signatory countries. In the context of our proposed Framework, involving counseling sessions for non-adherent patients, and the involvement of Civil Societies as treatment supporters in the communities, the number of patients needing to be contained should remain very small.

#### Other stakeholders

A key stakeholder group that must be considered in the framework are pharmaceutical companies. All first and second line drugs currently used for the treatment of drug-susceptible TB have been developed over 40 years ago, and are out of patent. National TB Programs therefore benefit from access to generic drugs of excellent quality at affordable prices. Unfortunately, counterfeit TB drugs are also available on the global market alongside the generic drugs [46]. Counterfeit drugs are often not pharmacologically adequate, and their circulation may greatly contribute to the spread and development of drug resistance [47]. Adherent patients may believe they are taking the appropriate regimen when in fact the drug dosage is too low or is uneven, which is effectively equivalent to skipping pills [48]. Therefore, public authorities through national legal regulations must require standards and more testing and control over the quality of the medicines that are imported in a country and take responsibility in banning counterfeit drugs of sub-optimal pharmacology. Simple and affordable tests are available to screen TB medications of substandard quality, a step that must be taken following the drug registration and tendering process in each country, before distribution to patients [49]. The legal framework could provide suggested penalties for pharmacists who dispense non-approved drugs from these companies producing counterfeit drugs that could lead to drug-resistance, if it is proven that the drugs were acquired illegally outside the national supply system.

It is important to note that two new compounds, bedaquiline and delamanid, have recently been developed and approved for treatment of MDR-TB [50,51]. Global patents covering pharmaceutical companies are governed by Trade Related Aspects of Intellectual Property Rights (TRIPS), which applies to all World Trade Organization (WTO) members. The TRIPS Agreement, enforced since 1995, introduced global minimum standards for protecting Intellectual Property Rights (IPR), including patents, and requires all WTO members to adapt their laws to the minimum standard of IPR protection. However, TRIPS also contains provisions that allow a certain degree of flexibility for countries to accommodate their own patent and IPR systems. Additionally, transition periods are granted, particularly to Least Developed Countries (LDCs). The transition periods have meant that pharmaceuticals or medicines patented before developing countries implemented their TRIPS obligations will not receive patent protection, and thus generic competition is possible. National TB Programs implementing treatment regimen that include either of those two new compounds will therefore also need to take steps to ensure the high quality of the drugs purchased and dispensed to patients, should they decide to procure generic versions of bedaquiline or delamanid.

The basis for including private sector companies in such an international framework convention is well proven. For example, a 2011 UN Report on Guiding Principles on Business and Human Rights stated that private companies must respect human rights and therefore:

“(a) Avoid causing or contributing to adverse human rights impacts through their own activities, and address such impacts when they occur; and (b) Seek to prevent or mitigate adverse human rights impacts that are directly linked to their operations, products or services by their business relationships, even if they have not contributed to those impacts [52].”

The obligations of the private sector was also a notable component in the FCTC, with private companies having to submit to testing of their products to comply with nationally defined standards for tobacco product content^l^, and comply with standards for product labels^m^ that attempt to discourage use. The example of the FCTC and UN recommendations on private sector responsibility for public health should be a component integrated into any international TB control framework.

Finally, public authorities and the government also have a shared responsibility to assure reliable and continuous access to quality health services. If public authorities fail to provide basic services, how can we expect the patient to adequately play their part? In the case of Kenya in 2010, measures were taken to limit the freedom of movement of non-adherent TB patients by incarceration, but the government failed to provide adequate structures to provide treatment in decent conditions. Indeed, these patients should have been isolated in a hospital setting instead of a prison, but that type of isolation facility was not available [53]. That is why it is important that the legal framework must hold public authorities and governments accountable. Strict guidelines as to what is appropriate for national TB laws to protect the patient and also ensure that communities are not threatened by the diseases, as well as mechanisms meant to strengthen the primary health care system, must be included within the framework.

#### Coordination mechanisms and ensuring adherence by each country

To facilitate the governance of the obligations under the TB control framework suggested above, a coordinating body of representatives from among the signatory countries should be set up, under the leadership of the WHO and the Stop TB Partnership. This body would be responsible for ensuring that signatory parties are following the regulations within the convention, and adopting national policies and guidelines in compliance. This body would also, in partnership with international organizations and like-minded supporters such as interested non-governmental and civil society organizations operating in the health and policy sectors, provide technical support as needed to state parties to assist in drafting legislation in keeping with the articles included within the convention.

As a part of their responsibilities, this proposed body would also review all cases of containment, benefiting both the patient and the states. It would provide a cumulative review of the processes and outcomes of such cases conducted by a specific country over the course of a year. This will be in addition to regular reports on each signatory parties achievements and challenges with respect to implementing the convention within their own borders. The oversight body will therefore ensure compliance with the various articles under the framework, and annually review the status with each signatory party, based on both a state’s annual report of activities under the framework, to be coordinated by the NTP in each country, as well as an external review as necessary.

Finally, an important recommendation for any such Convention must acknowledge that, while TB is a global disease, special considerations must be included for developing countries. A number of countries may not have the resources to implement fully such recommendations, particularly in the case of tracking patient follow-up, at least in the immediate term. Article 22 of the FCTC is relevant and should be translated into any global TB control framework: it recommended that technical assistance to each country, particularly those defined as “developing” or with “economies in transition”, be provided in order to help countries fulfill their obligations under the FCTC. Such recommendation has important repercussions for the ability of many countries in Africa and elsewhere to adhere to such a framework convention. While status as a developing country does not excuse any state from implementing the regulations such a TB framework would contain, it does acknowledge that additional technical capacity and assistance may be required.

#### Possible funding mechanisms

We envision that international funding streams relevant to TB, primarily the Global Fund, would make future TB grants contingent upon the country internalizing and implementing the recommendations within the framework convention, to act as a further incentive and ensure that the convention is being implemented within states, and to provide incentive for other states that may not have signed on to either sign the convention, or to still adopt the proposed regulations in their own countries. We also acknowledge that the Global Fund alone will not be able to cover the costs associated with the adequate implementation of the provisions defined in the Framework. Therefore, innovative funding strategies will need to be explored. One great example is UNITAID, which uses innovative financing to increase funding for greater access to treatments and diagnostics for HIV/AIDS, malaria and tuberculosis in low-income countries. Approximately half of UNITAID’s finances come from a levy on air tickets. A similar model could be designed, with a financing model based on a tax on, for example, mobile phones and tablets.

## Summary

The management of TB poses global health challenges but also ethical and legal ones. Though existing guidelines on TB prevention, care and control exist, further action is needed in order to make these guidelines internationally enforceable and justiciable, which is where a framework convention on TB control is needed. The FCTC can serve as an example for what such a convention can look like and how it can be implemented, being the first convention issued by the WHO. Such a framework needs to establish specific procedures and secure the rights and duties of all stakeholders.

Global management of TB is possible, if codified in such a framework that requires that state parties change policies and work together to control the disease both within their own borders and internationally. It is not just a framework that can and should be implemented in more developed countries, but will also, through international cooperation, directly impact the lives of those where the burden of TB is the highest. It takes the will of the state to implement measures to increase access to treatment, and it takes the contribution of the private sector, and a global cooperation, all as noted above, to implement this suggested framework. Successfully implementing policies that address patients’ rights and communities’ well-being can have positive implications for those affected by TB as well as other respiratory illnesses, and serve as a basis for other internationally agreed upon and enforced global health conventions and treaties to truly ensure the global right to health. Drafting a framework is not a panacea, but a necessary step. Public international law can be aspirational, and we understand that there is often tension between whether legal guidelines should reflect reality or an ideal. We are aiming for an ideal framework that sustainably impacts the global TB burden in measurable ways, and hope to start a productive discussion that will have tangible results benefiting TB patients and communities alike through this proposed Framework Convention on TB.

## Endnotes

^a^Article 2(k) of the WHO Constitution.

^b^Article 21 of the WHO Constitution.

^c^Article 63 of the WHO Constitution.

^d^Article 2(k) of the WHO Constitution.

^e^MDG tuberculosis control target: to halve the prevalence of tuberculosis disease and deaths between 1990 and 2015.

^f^55 recognized States.

^g^To separate ill persons who have a communicable disease from those who are healthy.

^h^Subject to confinement in prison.

^i^The TB-specific legislation is part of a Public Health Act of 1968.

^j^Home confinement is different from incarceration in a state-controlled institution, which is much harder to manage and much more likely to violate the human rights of an individual patient. The former method allows TB patients to choose to stay in a residential setting for their treatment.

^k^Originating in a hospital.

^l^Articles 9 and 10 of the Framework Convention on Tobacco Control.

^m^Article 11 of the Framework Convention on Tobacco Control.

## Abbreviations

CDC: Center for Disease Control and Prevention
CHW: Community Health Worker
DOTS: Directly Observed Therapy – Short course
FCTC: Framework Convention on Tobacco Control
GFATM: Global Fund to fight AIDS, Tuberculosis and Malaria
ICCPR: International Covenant on Civil and Political Rights
ICESCR: International Covenant on Economic, Social and Cultural Rights
MDR-TB: multidrug-resistant tuberculosis
NTP: National Tuberculosis Program
PEPFAR: President’s Emergency Plan for AIDS Relief
TB: Tuberculosis
UDHR: Universal Declaration of Human Rights
UN: United Nations
WHO: World Health Organization
XDR-TB: Extensively drug-resistant tuberculosis
